# Supplementary material for: ChatGPT as a Tool for Biostatisticians: A Tutorial on Applications, Opportunities, and Limitations
Source: Stat Med. 2025 Oct 23;44(23-24):e70263. doi: 10.1002/sim.70263 (PMC12548020; doi:10.1002/sim.70263)
Supplement: Supplementary file 1 — Data S1. Supporting Information. [file SIM-44-0-s001.zip › Supplementary Materials Additional.zip!/3.5_Causal_Inferece/3.5_Causal_Chat4.pdf]

Combining statistical and causal thinking_4 - Biostatistics Expert Role

Please clear memory.

I've cleared the memory of our past conversations. Let me know how I can assist you going forward!

Take the role of an expert biostatistician for this Chat

Understood. I'll take the role of an expert biostatistician. Feel free to share your question or project—whether it's about trial design, causal inference, survival analysis, missing data, model selection, or any other biostatistical topic.

I have data from a randomised clinical trial. I want to compare the efficacy of the experimental treatment with that of the control on overall survival in patients with non-small cell lung cancer. In this two-arm RCT, a considerable number of patients in the control arm switched to the experimental treatment after disease progression, while those in the experimental arm never switched.
My goal is to estimate the causal effect of sustained intake of the experimental treatment versus sustained intake of control on Overall survival, had the control group patients not been allowed to switch. Based on the information provided above, please define my target Estimand, following the guidance from the Estimand framework (ICH-E9 addendum).

Given your aim and the context of treatment switching, here's a clear definition of your **target estimand** based on the **ICH E9(R1) Estimand Framework**:

Estimand Definition

Treatment

- **Intervention of interest:** Sustained treatment with the experimental therapy from randomisation.
- **Comparator:** Sustained treatment with the control therapy from randomisation.
- Patients in both arms are considered to remain on their initially assigned treatment throughout follow-up, regardless of actual switching or discontinuation.

Population

- All randomised patients in the trial (intent-to-treat [ITT] population), specifically patients with non-small cell lung cancer (NSCLC) meeting eligibility criteria at baseline.

Variable (Endpoint)

- **Overall survival (OS):** Time from randomisation to death from any cause.

Intercurrent Events

- **Treatment switching** in the control arm is the key intercurrent event.
- You are choosing a **hypothetical strategy** to handle this intercurrent event: you aim to estimate what the outcome would have been **had no patients switched** from control to experimental treatment.

Other intercurrent events such as treatment discontinuation, subsequent therapies post-progression, or loss to follow-up can be handled via censoring, appropriate modelling, or aligned with the hypothetical strategy if they impact treatment intake.

Summary Measure

- The causal contrast of interest is the difference (or ratio) in overall survival distributions under the two sustained treatment strategies, e.g.:
  - **Hazard ratio** under a Cox model.
  - **Difference in restricted mean survival time (RMST)** at a specified time point.
  - **Survival probability difference** at fixed follow-up times (e.g., 12 or 24 months).

Summary of the Target Estimand

> *In patients with NSCLC enrolled in this RCT, the difference in overall survival between a strategy in which all patients received sustained treatment with the experimental therapy versus a strategy in which all patients received sustained treatment with the control therapy, assuming that no patients in the control arm switched to the experimental treatment after disease progression (hypothetical strategy).*

Let me know if you'd like to discuss methods for estimation (e.g., IPCW, RPSFT, G-estimation) or sensitivity analysis for assumptions under this estimand.

The switching behaviour is affected by a set of different baseline and time-varying covariates. Here, I want to assume that the time-varying confounders that influence the switching behaviour and Overall survival are also themselves affected by prior treatment intake.
For e.g. ECOG score might not only affect whether patients should switch treatment or not, but its value might also be affected by prior treatment intake reflected by the switching behaviour.
In this case, the analytical problem becomes more complex. Can you explain?

Yes, you are describing a classic **time-varying confounding problem with treatment-confounder feedback**, which complicates the estimation of causal effects. Here's a structured explanation tailored to your setting:

---

1. The Complexity: Treatment-Confounder Feedback

In your trial:

- Patients in the **control arm may switch** to the experimental treatment after progression.
- This **switching decision is influenced by time-varying covariates** (e.g. ECOG score, tumour progression, target lesion size).
- Crucially, these **time-varying covariates are themselves influenced by prior treatment** (e.g. ECOG score might improve or deteriorate based on whether a patient has started receiving experimental therapy).

Treatment-confounder feedback arises because:

- Time-varying covariates **predict future treatment decisions** (e.g. switching).
- But they are also **affected by past treatment** decisions (e.g. past switching).
- Therefore, **standard regression approaches (like Cox with time-dependent covariates)** will be **biased**, because they incorrectly adjust for post-treatment variables that are on the causal pathway.

---

2. Why This Breaks Standard Methods

Standard methods (e.g., time-dependent Cox regression, stratification, or standard IPW) fail because:

- They **condition on variables affected by prior treatment**.
- This can **block causal pathways** or **open collider paths**, introducing bias.
- You can **bias away part of the true treatment effect** or **introduce spurious associations**.

---

3. Appropriate Analytical Methods

To correctly estimate the causal effect of **sustained treatment strategies** in the presence of **treatment-confounder feedback**, you need **G-methods** developed for such settings:

(a) Inverse Probability of Treatment Weighting (IPTW) of Marginal Structural Models (MSMs)

- Construct weights that reflect the **probability of receiving treatment (switching) conditional on past covariates and treatment history**.

- These weights **remove time-varying confounding** by balancing observed covariates **without conditioning on them directly** in the outcome model.
- Can estimate marginal survival curves or hazard ratios under sustained strategies.

(b) G-Estimation of Structural Nested Models

- Targets **treatment effects conditional on potential outcomes**.
- Often more efficient than IPTW but more complex to implement and interpret.

(c) G-Formula (Parametric or Monte Carlo)

- Models the full joint distribution of time-varying covariates, treatment, and outcome.
- Simulates counterfactual outcomes under different treatment regimens (e.g. no switching).
- Very flexible but requires strong modelling assumptions.

---

4. Summary of the Causal Structure

You have the following **causal DAG** features:

```lua
L0 → A0 → L1 → A1 → L2 → Y
  ↘       ↘       ↘
   ↘-------→-------→
```

- **L0, L1, L2** = Time-varying confounders (e.g. ECOG, progression, tumour burden).
- **A0, A1** = Treatment received at each time (initial assignment, then switching).
- **Y** = Overall survival.

Where:

- **L_t depends on A_{t-1}**
- **A_t depends on L_t**
- **Y depends on both A_t and L_t**

This feedback loop between treatment and confounders violates the **"no time-varying confounding"** assumption required by standard models, hence the need for **G-methods**.

Next Steps

You'll need to:

1. **Specify the target causal contrast** under a sustained treatment regime (as we did).
2. **Construct a time-varying dataset** with repeated measurements of covariates, treatment, and survival time.

3. **Model treatment switching (propensity scores) and censoring (if informative)** to construct weights.
4. **Estimate effects using MSMs, G-formula, or G-estimation**, depending on data and software.

Let me know if you'd like help selecting a method or structuring the data for such an analysis.

Depict our research problem in a DAG

Here's a causal Directed Acyclic Graph (DAG) that captures the core structure of your research problem—treatment switching in a randomised controlled trial with time-varying confounding **affected by prior treatment**.

Causal DAG: Treatment Switching with Time-varying Confounding

```lua
   L0
   |\
   | \
   v  \
  A0   \
   |     \
   v      v
   L1 --> A1
    |      |
    v      v
   L2 --> A2
    |      |
    v      v
    ...   ...
           |
           v
           Y
```

Node Definitions

- **L0**: Baseline covariates (e.g. age, ECOG at baseline, disease burden, stratification factors).
- **At**: Treatment at time *t*.
  - **A0**: Initial randomisation (experimental vs control).
  - **A1, A2, ...**: Switching behaviour / treatment actually received over time (e.g. from control to experimental).
- **Lt**: Time-varying confounders (e.g. ECOG score at time *t*, tumour size, disease progression).
- **Y**: Outcome (Overall Survival).

Key Arrows and Dependencies

- **L0 → A0**: Randomisation depends on L0 (stratified randomisation or just pre-randomisation knowledge).

- **L0 → Lt**: Baseline covariates affect later health status (e.g. tumour burden affects progression).
- **A0 → Lt**: Initial treatment affects patient condition over time (e.g. treatment improves ECOG).
- **Lt → At+1**: Patient condition determines future treatment decisions (e.g. triggers switching).
- **At → Lt+1**: Treatment influences subsequent health status (treatment-confounder feedback).
- **At, Lt → Y**: Both treatment and time-varying covariates affect overall survival.

Implications of This DAG

- You cannot adjust for **Lt** in standard outcome models because it's affected by prior **At**.
- There's **treatment-confounder feedback**, which biases standard Cox models or naïve adjustment methods.
- To estimate the causal effect of sustained treatment strategies (e.g. always remain on assigned treatment), you need **G-methods** like **IPTW of marginal structural models**, **G-formula**, or **structural nested models**.

Would you like a visual version of this DAG as a figure (e.g. for a paper or presentation)?

Yes go ahead

Image created

I would like to apply the inverse probability of censoring weighting (IPCW) approach to address the bias introduced by switching following the methodology discussed in the following paper: Robins JM, Finkelstein DM. Correcting for noncompliance and dependent censoring in an AIDS clinical trial with inverse probability of censoring weighted (IPCW) log-rank tests. Biometrics. 2000;56(3):779–88.
Briefly summarise the key points from this paper

Certainly. Here's a concise summary of the key points from the influential paper by **Robins and Finkelstein (2000)** on the **Inverse Probability of Censoring Weighting (IPCW) log-rank test**, which is highly relevant to your setting:

Paper Summary: Robins & Finkelstein (2000)

"Correcting for Noncompliance and Dependent Censoring in an AIDS Clinical Trial with IPCW Log-Rank Tests"
Biometrics. 2000;56(3):779–788

Context & Problem

- The paper addresses **treatment noncompliance** and **treatment switching** in randomised clinical trials.
- Specifically, it tackles **dependent (informative) censoring**, where the decision to switch treatments (or deviate from protocol) is **related to prognostic factors** that also affect the outcome (e.g. survival).

Objective

- To develop a method that yields **unbiased causal estimates of treatment effects** under a **hypothetical strategy**: what would have happened if patients had adhered to their originally assigned treatment throughout follow-up.

Key Concepts and Methodology

1. Hypothetical Estimand

- The goal is to estimate survival outcomes **under full compliance with randomised treatment** (i.e. sustained treatment without switching).
- This is framed as a **causal contrast** between sustained treatment regimes.

2. Dependent Censoring

- Patients who **switch treatments** (e.g. control → experimental) are **administratively censored** at the time of switching.
- However, this censoring is **informative**, as switching may depend on time-varying health status and prognosis.

3. IPCW Approach

- Robins & Finkelstein propose **Inverse Probability of Censoring Weights (IPCW)** to correct for the bias introduced by dependent censoring.
- Each individual is weighted by the **inverse of the estimated probability of remaining uncensored up to each time point**, given their covariate and treatment history.

4. Weighted Log-Rank Test

- They use IPCW to construct a **weighted log-rank test**, which tests the null hypothesis of **no treatment effect under full adherence**.
- This test is robust to noncompliance and allows for valid inference about the causal effect of sustained treatment assignment.

Assumptions

The method relies on key assumptions:

1. **No unmeasured confounding of the censoring mechanism** (i.e. switching depends only on observed covariates and treatment history).
2. **Correct specification of the model** used to estimate the censoring probabilities.
3. **Positivity**: Each patient has a non-zero probability of remaining uncensored up to the time they are censored.

Implications for Your Study

You can adopt this IPCW approach in your trial where:

- **Control patients switch to experimental therapy**, introducing dependent censoring.
- You wish to estimate **causal effects under a hypothetical strategy of no switching**.
- You would:
  - Censor patients at time of switching.
  - Estimate **weights** based on the probability of not having switched by time *t*, conditional on observed covariate history.
  - Fit a **weighted survival model** (e.g., log-rank test or Cox model) using these IPCW weights.

Let me know if you'd like help implementing IPCW in your setting (e.g., how to structure the data, specify models for the weights, or apply the weights in survival analysis).

Could you elaborate more on the research question Robins and Finkelstein were trying to answer in their paper, mentioning the comparator arms, possible intercurrent events, outcome definition and population under study.

Certainly. Here's a detailed breakdown of the **research question** that **Robins and Finkelstein (2000)** aimed to address in their landmark paper, using the ICH E9(R1) estimand framework terminology where applicable.

Study Context and Background

The paper reanalyses data from an **AIDS clinical trial** known as the **AIDS Clinical Trial Group Protocol 019 (ACTG 019)**.

- **Objective of ACTG 019**: Compare the effect of different antiretroviral regimens on disease progression or death in HIV-infected individuals with low CD4 counts.

Estimand Framework Breakdown

1. Treatment

- **Intervention**: *Immediate initiation* of **zidovudine (AZT)** at 500 mg/day.
- **Comparator**: *Deferred initiation* (i.e. **placebo** initially, with AZT allowed after clinical progression or a drop in CD4 count).

This design created an **intent-to-treat randomisation**, but **switching (noncompliance)** was common in the deferred arm after clinical events.

2. Population

- HIV-infected patients enrolled in ACTG 019 with **baseline CD4 counts between 200 and 500/mm^3**.
- These patients were considered at risk for AIDS progression but were not yet symptomatic.

3. Variable (Outcome)

- A **composite clinical outcome**:
  - Time from randomisation to **AIDS-defining event** or **death**.

4. Intercurrent Events

The paper explicitly addresses **intercurrent events** that interfere with the interpretation of the treatment effect:

- **Treatment switching**: Many patients in the placebo (deferred AZT) arm **switched to AZT** upon progression or low CD4 count, violating sustained treatment strategies.
- **Treatment noncompliance**: Patients did not always adhere to the assigned treatment.

The authors adopted a **hypothetical strategy** to address these intercurrent events:

- They asked: *What would the survival curves have looked like if all patients had adhered to their originally assigned treatment (sustained placebo vs sustained AZT)?*

To estimate this, they **censored patients at the time of deviation** (e.g. switching from placebo to AZT) and corrected for the induced selection bias using IPCW.

5. Summary Measure

- The primary comparison was made using a **weighted log-rank test** to compare survival under **sustained placebo vs sustained AZT**, had there been **full adherence**.
- The method provides a **causal contrast** between these sustained strategies, rather than an ITT effect.

Research Question in Plain Terms

> *In HIV-positive patients with moderate immunosuppression, what is the effect of sustained treatment with AZT (500 mg/day) from randomisation, compared with sustained placebo (no AZT), on time to AIDS progression or death—under the hypothetical scenario where all patients adhered to their initially assigned treatment and did not switch?*

This question contrasts with the **intention-to-treat (ITT) analysis**, which mixes treatment strategies due to switching and noncompliance, potentially underestimating the true effect of sustained AZT.

Robins and Finkelstein's IPCW approach enabled estimation of the **causal effect under full adherence** using **observational tools within a randomised trial**, correcting for **informative censoring** due to treatment switching.

Let me know if you'd like to translate this structure into your own NSCLC trial context for formal reporting.

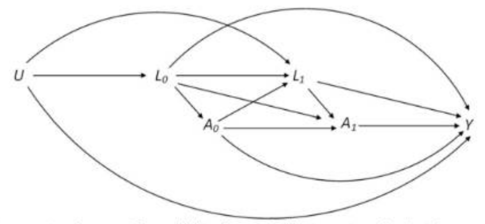

Fig. 1 Graphical representations of our research focus and the bias of naive methods for time-varying confounding in the presence of treatment-confounder feedback: This Directed acyclic graph (DAG) represents the post-progression period for patients in the control arm. Following disease progression, the decision as to whether or not a patient in the control group continues to take its treatment (A_0) or (A_1) depends on predictive factors measured at or before the time of disease progression (L_0). Again, we assume that treatment intake (A_0) affects outcome (Y) and influences a set of TVCs at a later time-point (L_1). These covariates are also assumed to impact the clinician's decision as to whether or not a patient should subsequently change treatments (A_1) introducing time-varying confounding and treatment-confounder feedback through L_1

Attached, I provide you with a directed acyclic graph (DAG) that represents our research problem.
To simplify this DAG and take into consideration this deterministic information provided below, I restrict my DAG to the post-progression period for patients in the control arm only and consider two follow-up time points, 1 & 2.
Here A(k) is deterministically 0 if A(k-1)=0 AND L(k-1) (reflecting progression status)=0 (no progression).
Following disease progression, the decision as to whether or not a patient in the control group continues to take their treatment (A0 or A1) depends on predictive factors measured at or before the time of disease progression (L0).
Again, we assume that treatment intake (A0) affects outcome (Y) and influences a set of TVCS at a later time-point (L1).
These covariates are also assumed to impact the clinician's decision as to whether or not a patient should subsequently change treatments (A1), introducing time-varying confounding and treatment confounder feedback through L1.
I will emphasise again that in our case scenario, patients in the control group do not switch unless they progress, and all patients in the experimental group never switch.
I still want to estimate in my outcome model the causal effects of sustained treatment interventions (always taking control versus always taking experimental) under a hypothetical strategy where control patients do not switch treatment.
I want you to use this DAG as a basis for performing the appropriate IPCW analyses and provide me with the analysis R-script.
You may assume that the dataset is in long format, with variables: id, time (in months and increases with increments of 1), death indicator, switching indicator, baseline covariates
repeated over time points, time-varying covariates at each time pt.

DAG Representing Post-Progression Period (Control Arm)

DAG Representing Post-Progression Period (Control Arm)

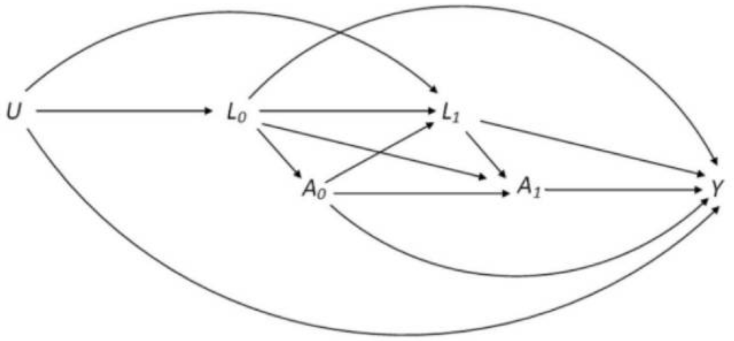

Fig. 1 Graphical representations of our research focus and the bias of naive methods for time-varying confounding in the presence of treatment-confounder feedback: This Directed acyclic graph (DAG) represents the post-progression period for patients in the control arm. Following disease progression, the decision as to whether or not a patient in the control group continues to take its treatment (A_0) or (A_1) depends on predictive factors measured at or before the time of disease progression (L_0). Again, we assume that treatment intake (A_0) affects outcome (Y) and influences a set of TVCs at a later time-point (L_1). These covariates are also assumed to impact the clinician's decision as to whether or not a patient should subsequently change treatments (A_1) introducing time-varying confounding and treatment-confounder feedback through L_1

Based on the DAG you provided and the IPCW framework, here is an R script outline tailored to your longitudinal dataset (in long format) to implement **IPCW for estimating the causal effect of sustained control versus sustained experimental treatment**, assuming no switching.

🔧 Assumptions:

- Patients switch only after progression.
- Switching creates **dependent censoring**.
- You model the **probability of not switching**, i.e., remaining uncensored under a hypothetical no-switching regime.

✅ R Script for IPCW (Simplified and Adaptable)

```r

# Load required packages
library(survival)
library(dplyr)
library(ipw)

# Step 1: Define the data structure
# Assumes long format with variables:
# id, time, death (0/1), switch (0/1), baseline_cov1, baseline_cov2, tvc1, tvc2, ...

# Step 2: Define a censoring indicator (1 = censored due to switching)
data <- data %>%
  mutate(censor = ifelse(switch == 1, 1, 0))

# Step 3: Fit the model for censoring (probability of NOT switching)
# Use pooled logistic regression for time-dependent censoring model
# (You can include time-splines or factor(time) for flexibility)
censor_model <- glm(censor == 0 ~ time + baseline_cov1 + baseline_cov2 + tvc1 + tvc2,
                    data = data,
                    family = binomial())

# Step 4: Predict probabilities of remaining uncensored
data <- data %>%
  mutate(p_uncensored = predict(censor_model, type = "response"))

# Step 5: Calculate inverse probability weights
```

```
data <- data %>%
  group_by(id) %>%
  arrange(time) %>%
  mutate(ipcw_weight = cumprod(p_uncensored)) %>%
  ungroup()

# Step 6: Create the survival object
# Note: Ensure 'death' is 1 at the true event time, 0 otherwise
surv_data <- data %>%
  group_by(id) %>%
  summarise(
    time = max(time),
    death = max(death),
    weight = last(ipcw_weight),
    treatment = first(treatment)  # 0 = control, 1 = experimental
  )

# Step 7: Fit weighted Cox model (or weighted survival curve)
cox_model <- coxph(Surv(time, death) ~ treatment, weights = weight, data = surv_data)
summary(cox_model)

# Alternatively, estimate weighted KM curves
km_fit <- survfit(Surv(time, death) ~ treatment, weights = weight, data = surv_data)
plot(km_fit, col = c("blue", "red"), lty = 1:2)
legend("bottomleft", legend = c("Control", "Experimental"), col = c("blue", "red"), lty = 1:2)
```

📌 Notes for Adaptation

- You may want to stratify the censoring model by time (e.g. `` `factor(time)` ``), or use splines for smoother modelling.
- Baseline and time-varying covariates in the censoring model should reflect the **common causes of switching and death**.
- Time-varying covariates must be updated correctly across rows per patient.
- Assumes **switching time = censoring time** for estimating the hypothetical estimand.

Would you like help tailoring this code to your specific covariates or using stabilized weights and truncation?
